# The Neural Basis of Empathy and Empathic Behavior in the Context of Chronic Trauma

**DOI:** 10.3389/fpsyt.2019.00562

**Published:** 2019-08-16

**Authors:** Jonathan Levy, Karen Yirmiya, Abraham Goldstein, Ruth Feldman

**Affiliations:** ^1^Baruch Ivcher School of Psychology, Interdisciplinary Center Herzliya, Herzliya, Israel; ^2^Department of Psychology and the Gonda Brain Center, Bar-Ilan University, Ramat Gan, Israel; ^3^Child Study Center, School of Medicine, Yale University, New Haven, CT, United States

**Keywords:** social brain, parent-child relationship, early trauma, magnetoencephalography, empathy

## Abstract

**Background:** Accumulating evidence in social neuroscience suggests that mature human empathy relies on the integration of two types of processes: a lower-order process mainly tapping into automatic and sensory mechanisms and a higher-order process involving affect and cognition and modulated by top-down control. Studies have also indicated that neural measures of empathy often correlate with behavioral measures of empathy. Yet, little is known on the effects of chronic trauma on the neural and behavioral indices of empathy and the associations among them.

**Methods:** Mothers exposed to chronic war-related trauma and nonexposed controls (N = 88, N = 41 war-exposed) underwent magnetoencephalography (MEG) while watching stimuli depicting vicarious emotional distress. Maternal empathic behavior was assessed during mother–child interaction involving a joint task.

**Results:** Empathy-evoking vignettes elicited response in alpha rhythms in a network involving both sensorimotor and viceromotor (anterior insula) regions, suggesting integration of the sensory and affective components of empathy. Whereas exposure to chronic stress did not affect the level of neural activations in this network, it reduced maternal empathic behavior. Furthermore, trauma exposure impaired the coherence of brain and behavior; only among controls, but not among trauma-exposed mothers, the neural basis of empathy was predicted by maternal empathic behavior.

**Conclusions:** Chronic stress takes a toll on the mother’s empathic ability and indirectly impacts the neural basis of empathy by disrupting the coherence of brain and behavior.

## Introduction

Empathy is a multifaceted psychological construct that plays a key role in social life and enables humans to feel and understand each other and form social groups and cultural communities. The neuroscience of empathy, an emerging field of research, has been informative in defining the multiple facets of empathy and the associations between its neural and behavioral manifestations (1). One aspect which has been repeatedly observed in various neuroimaging studies is that human empathy relies on the integration of two types of processes; a lower-order process mainly tapping into automatic and sensory mechanisms and a higher-order process involving affect and cognition and top-down control. Importantly, both the automatic and higher-order mechanisms are needed for the expression of mature, full-blown empathy (2, 3). Functional neuroimaging studies have shown that mature human empathy integrates these two neural components and relies on the involvement of both sensorimotor (SM) and frontal viceromotor regions, mainly the anterior insula (AI). Such dual-system activations have been interpreted to reflect the integration of the lower-order, automatic, and sensory aspect of empathy with the higher-order, cognitive, and top-down aspect (2, 4–6). In parallel, EEG and magnetoencephalography (MEG) studies showed that the alpha, or *Mu* rhythm (alpha rhythms in sensory–motor areas) conveys a reliable marker of empathy (7–11). Consistent with these findings, we have shown in a large-scale MEG study of children, adolescents, and adults that whereas children and adolescents’ response to others’ pain implicated alpha rhythms in sensory-motor regions, only in adulthood did participants exhibit both sensory–motor and higher-order activations in viceromotor areas that support interoceptive sensitivity of one’s own bodily milieu in the service of other-focused empathy (12). Taken together, these findings suggest that mature human empathy is underpinned by the SM-AI network that sustains full-blown adult empathy *via* the alpha rhythm.

Studies on the brain basis of empathy typically examine associations between neural activations in paradigms that call for empathic response and behavioral or self-reported indices of empathy (2, 13–16). Several studies revealed a robust link between measures of empathy in the brain and measures of observed social behavior, including empathy and synchrony or negative correlations between the neural empathic response and hostile behavior (11, 17). Yet, other studies failed to report such links and found no associations between the neural basis of empathy and empathic behavior. One explanation for such lack of correlations may relate to the heterogeneity of participants: it is possible that brain–behavior links exist only for certain groups of individuals but not for others. While this hypothesis has not been studied in depth, prior evidence lends support to this assumption. For instance, in a study on the neural basis of attachment, we found that among synchronous mothers, associations emerged between neural activations in key nodes of the maternal brain, oxytocin levels, and the mother’s attuned parenting behavior; however, such links were not found for intrusive mothers, indicating that coherence among the neural and behavioral indices of social functions may index greater maturity and more optimal functioning (18). Similarly, children who experienced more empathic and synchronous parenting showed a greater coherence of theta, alpha, beta, and gamma rhythms across the social brain, including the superior temporal sulcus (STS)/superior temporal gyrus (STG) and AI and greater correlations of brain and behavior (19).

These findings raise the interesting assumption that coherence between activations in the social brain and observed social behavior may serve as a marker of greater maturity and functionality (20). This novel assumption is supported by several lines of evidence from various methods and samples. First, a previous functional magnetic resonance imaging (fMRI) study suggested that mothers tune their child’s brain *via* behavior-based processes (21), while another fMRI study demonstrated that the integrity of empathic networks in the parental brain shape children’s long-term behavior (22). A MEG study found that when mothers and children engaged in synchronous interactions, they also exhibited brain-to-brain synchrony which was tightly connected to their behavior, particularly to mothers’ empathic behavior (23). A recent transcranial magnetic stimulation (TMS) study demonstrated a causal relationship between empathic neural response and prosocial behavior (24). Finally, animal studies also conveyed a clear coordination of mother and offspring’s physiological systems with bottom-up behavioral processes (25, 26). Among the conditions that may disrupt the expression of brain–behavior correlations, particularly in the social brain, are chronic stress and prolonged trauma. Prolonged exposure to trauma has long been known to increase psychopathology and suicidal behavior (27), reduce social adaptation, and impair brain functioning (28). Prolonged exposure to trauma, particularly during early development, has long been known to increase psychopathology, and early life stress was found to explain nearly 32% of psychiatric disorders (29). In an earlier study of the current research cohort, over 80% of children exposed to early and chronic trauma displayed a full-blown Axis I disorder at some point in their childhood (30), and exposed mothers were found to have higher depression, anxiety, and posttraumatic stress disorder (PTSD) symptoms compared with controls (31). In addition to such deficits, studies have repeatedly shown that individuals exposed to early trauma exhibit impaired empathy (32–34) and abnormal neural functioning of social functions including response to negative emotional stimuli (35, 36). Chronic stress and trauma also impair the neural basis of empathy. For instance, a MEG study showed that veterans exposed to wartime trauma exhibited disrupted neural empathic response to others’ pain as expressed by the alpha rhythm (37). In the context of parenting, another MEG study similarly showed disruptions in the neural basis of empathy among adolescents exposed to maternal depression throughout their first years of life and these disruptions were again found in the alpha rhythm (17). Thus, while no prior study examined the effects of chronic trauma on brain–behavior coherence in relation to the empathic brain, the aforementioned standard error (SEM) studies suggest that diminished coherence may be one result of chronic trauma exposure.

**Figure 1:**
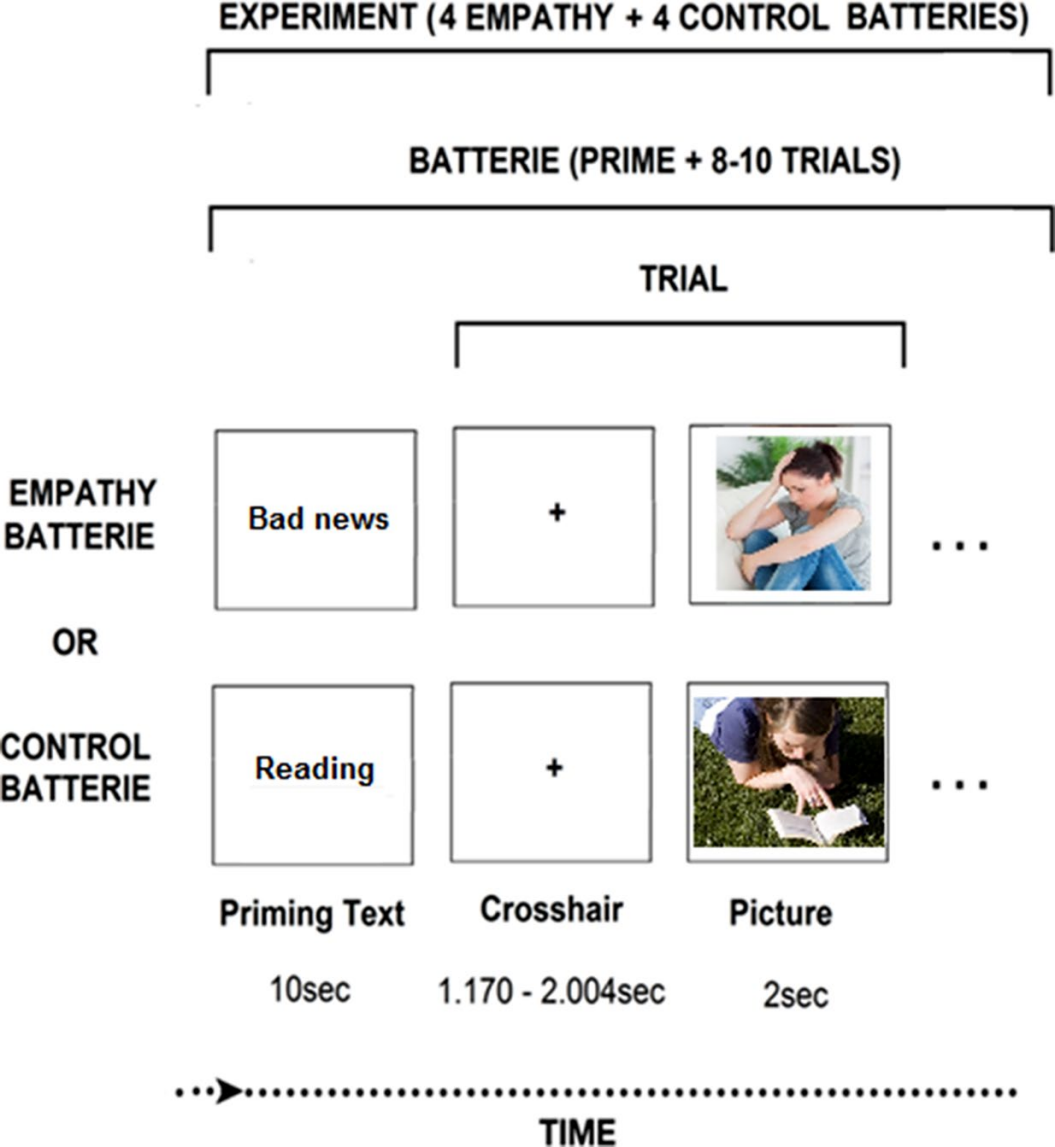
Neuroimaging experimental design. The “bad news” photo: Wavebreak Media Ltd © 123RF.com. The “reading” picture: FreeImages.com/Ryan Day.

In light of the above, the current study examined whether prolonged exposure to trauma may either directly impact the empathic brain, or alternatively, indirectly affect the associations between the neural and behavioral markers of empathy. Previous research has shown that exposure to chronic stress impairs the neural foundation of empathy and its behavioral manifestations (35, 36, 38–40), but the association between the two has not been tested thus far. Further, we were interested to test whether empathic behavior in the context of parenting could predict neural empathic response based on our previous studies which indicated that empathic parenting, particularly affectively synchronized behavior that is tuned to the child’s state and communications, is an individually stable maternal orientation when measured repeatedly from infancy and through adolescence and, moreover, such synchronized maternal behavior predicts the neural empathic response (17, 23, 41, 42). To probe the impact of chronic stress on the neural basis of empathy, we used an empathy paradigm which simulates empathy to vicarious affective stress (**Figure 1**). This paradigm has shown to yield activations containing both the SM and AI components of empathy (13).

We utilized a sample of mothers who were exposed to chronic war-related trauma as representing a condition of mass trauma. Such trauma is defined by the exposure of large populations to the same natural disaster or war/violence condition at the same time point (43). One of the main characteristic of such trauma is that typically the whole family is exposed to the trauma together. As a result, mothers experience both the stress of being exposed to chronic war themselves, as well as raising their children in an atmosphere of constant fear and handing their children’s worries and stress responses. Children depend on their cargivers to supply protection, security, and emotional regulation, especialy following trauma exposure (44). It is thus of imprtance to investigate the foundations of maternal sensitivity, support, and empathic behavior in the context of chronic stress. Three hypotheses were proposed. First, we expected trauma-exposed mothers to show lower behavioral empathy during interaction with their children. Second, we expected to see alterations in the neural basis of empathy in mothers living in a context of chronic trauma. Finally, we expected to see group differences in the connection of brain and behavior, such that only among the nonexposed mothers there will be correlations between greater neural activations to others’ emotional distress and more empathic parenting, whereas in mothers exposed to chronic stress there will be a disconnect between brain and behavior.

## Methods and Materials

### Participants

Participants were mothers of children who were part of a longitudinal study on the effects of war exposure on mothers and children. Between the years 2004 and 2005, we recruited 232 mothers (M ± SD 31.27 ± 5.55 years) and young children (M ± SD 2.76 ± 0.91years) in two groups; trauma exposed and controls. The trauma-exposed group included 148 families (148 mothers and 148 infants) from Sderot, a town in the south of Israel located 10 km from the Gaza border. This area has suffered for the past 20 years from chronic rockets and missiles attacks, a few wars, and military operations. The second group included 84 nonexposed control families from other areas in the center of Israel. The two groups were matched demographically in terms of age, gender, birth order, parental age and education, maternal employment, and marital status and screened for other trauma, and we included participants who were not physically or mentally handicapped (e.g., severe autism, mental retardation) [for more details, see (45)].

The current study presents data from the fourth stage of this longitudinal study, when children were at preadolescence (11–13 years). At that longitudinal stage, compatibility with MEG scanning was added to the inclusion criteria: this mainly required that participants were metal free (e.g., free of tooth-bracelets, metallic implants) and were not pregnant. Eighty-eight mothers^1^ participated in MEG scanning (M ± SD 40.37 ± 5.26 years) and their children were now in preadolescence (M ± SD 11.65 ± 1.25); 41 of mothers were war exposed. Of 107 mothers participating in T4, 19 did not complete the MEG experiment: 8 were MEG incompatible, 5 declined the MEG part, 4 had poor MEG signal, and 2 were pregnant. The study was approved by the Ethics Committee at Bar Ilan University. Written informed consent was obtained from all participants. Experiments were performed in accordance with ethical guidelines.

### Procedure and Measures

***MEG Paradigm***. During MEG scanning, we employed an empathy task contrasting situations where same-age targets were in distress (DS) versus non-distress(no-DS) and mothers were asked to take the targets’ perspective and put themselves in “the other person’s shoes” (13). This experimental contrast involves sensitivity to vicarious DS and activates both sensory and affective components of empathy (13). We created pool of 128 stimuli (photos in uniform size: 300 × 225 pixels) half depicting DS/anxiety situations and half neutral. Distress situations described typical anxiety-promoting (social exclusion, exam stress) versus nondistressing (shoe-lacing, reading) events in preadolescents’ lives. Stimuli were piloted until the final 128 stimuli were each validated by independent raters (n = 21). Stimuli’s affective valence (1-very negative, 2-negative, 3-neutral, 4-positive, 5-very positive) was rated as neutral (M ± SD 3.04 ± 0.25) and negative (M ± SD 1.95 ± .28) for the no-DS and DS stimuli, respectively, with a statistically significant difference (*P* = 6.21 * 10^−47^) between categories. Stimuli’s affective arousal (1-very low to 5-very high) was rated as low (M ± SD 2.05 ± 0.33) and high (M ± SD 3.83 ± .42) for the no-DS and DS stimuli, respectively, with a statistically significant difference (*P* = 2.37 * 10^−53^) between categories. Finally, stimuli were matched for physical parameters, including complexity, contrast, and luminance, resulting in no statistically significant difference (*P* > .35) on any of these parameters. Photos were presented in blocks preceded by a contextual sentence, generically describing the situation in the ensuing photo (e.g., “this person heard that his friends plan to exclude him”, “this person reads about the history of Sweden”). Sentences were designed to consist of M ± SD 9.07 ± 1.14 words and M ± SD 43.64 ± 5.10 characters long, with no statistically significant difference (*P* > .3) in length between categories. Paradigm was programmed and operated using E-Prime^®^ 2 software (Psychology Software Tools Incorporated).

***Imaging Session.*** Participants laid in supine position inside the MEG system while facing a screen projecting the stimuli in the center of gray background of 20-inch monitor at distance of 50 cm. Participants were told to take the targets’ perspective and to imagine how he/she felt in that situation. Fourteen blocks consisted each of a contextual sentence describing the situation followed by 8–10 photos depicting different individuals in that situation. Sentences and photos were presented for 10 s and 2 s respectively. The interstimulus interval was jittered for 1.170–2.004 s and the interblock interval was jittered for 4.170 s–5.004 s. Participants were trained by watching two exemplar blocks and instructed to remain relaxed and not move their head or body and to pay attention to the events depicted in the photos. Movements were visually monitored by the experimenter *via* a camera, and by five coils attached to the participants’ scalp to record head position relative to the sensor array.

***MEG Recordings and Data Preprocessing*** **.** We recorded ongoing brain activity (sampling rate, 1,017 Hz, online 1–400 Hz band-pass filter) using a whole-head 248-channel magnetometer array (4-D Neuroimaging, Magnes^®^ 3600 WH) inside magnetically shielded room. Reference coils located approximately 30 cm above the head, oriented by *x*, *y*, and *z* axes enabled removal of environmental noise. Head shape underwent manual digitization (Polhemus FASTRAK^®^ digitizer). External noise (e.g., power-line, mechanical vibrations) and heartbeat artifacts were removed from the data using a predesigned algorithm for that purpose (46) and trials containing muscle artifacts and signal jumps were rejected from further analysis by visual inspection. We analyzed data of 2,000 ms epochs including baseline period of 700 ms filtered in the 1–200 Hz range with 10 sec padding and then resampled to 400 Hz.

***Maternal Behavior.*** *Maternal empathic behavior* was observed during a mother–child interaction, which took place about 20 min prior to the MEG paradigm, and included a joint task. The interaction was videotaped and coded offline using the Coding Interactive Behavior Manual (CIB) (47). The CIB is a well-validated system for coding social behavior, extensively used across cultures and psychiatric conditions from infancy to adulthood (48). The CIB includes multiple scales coded from 1 (low) to 5 (high), which are averaged into theoretically determined constructs. Coding was conducted by trained coders who were blind to any other information, and reliability on 20% of the interactions exceeded 90% on all codes (k = 0.82, range = 0.78–0.95). *The maternal empathic behavior* construct included the following scales: emotional empathy, cognitive empathy, behavioral empathy, acknowledgement/recognition of the child affect and communication, expansion of the child’s statements, and containment of the child’s DS and high arousal (Cronbach’s α = .92). From the 88 mothers who participated in the current study, eight dyads did not have a filmed interaction due to technical issues.

***MEG Analyses.*** We analyzed data in alignment to stimulus onset and then averaged the power estimates across tapers. We performed analyses using MATLAB 11 (MathWorks^®^, Natick, MA, USA) and the FieldTrip software toolbox (49). To calculate induced oscillatory activity in the alpha band, a Hanning taper, applied to each epoch of the 248-sensor data yielded the FFT for short sliding time windows of 0.5 sec in the 6–15 Hz frequency range, resulting in spectral resolution of 2 Hz. For source localization, we built a single shell brain model based on MNI post-puberty template brain (50), modified to fit each subject’s digitized head shape using SPM8 (51). The subject’s brain volume was then divided into a regular grid. The grid positions were obtained by a linear transformation of the grid positions in a canonical 1-cm grid. This procedure facilitates the group analysis because no spatial interpolation of the volumes of reconstructed activity is required. For each grid position, spatial filters were reconstructed in the aim of optimally passing activity from the location of interest, while suppressing activity which was not of interest. The spatial filter which we applied relies on partial canonical correlations (49, 52) and its cross-spectral density (CSD) matrix was computed between all MEG sensor pairs from the Fourier transforms of the tapered data epochs at the statistically significant time–frequency sensor pattern (**Figure 3**, left upper panel).

***Behavioral Analyses.*** T-tests were used to compare brain and behavior variables between exposed and controls. Next, Pearson correlations assessed relationships between maternal brain and behavioral variables. In order to test our hypothesis regarding the different association between behavior and brain in the two groups, contemporary practices of simple linear moderation model by Hayes (53) was adopted. To estimate the conditional effect of the independent variable maternal behavior (X), on the outcome variable SM-AI (Y), with chronic stress exposure included as a moderator (M), the PROCESS macro for SPSS (v. 2.1.3.2) Model 1 was used (54). Exposure was dummy coded, with the control group given a value of “0” and the exposed group a value of “1”. Maternal behavior and exposure were mean centered to facilitate the interpretation of the simple and interaction effects (54, 55). Following, the maternal behavior, exposure, and maternal brain activity variables were standardized in order to compute the standardized effects. The product term between mean centered maternal behavior scores and the exposure group was computed to test for the maternal behavior-by-group interaction. The group, maternal behavior, and group-by-maternal behavior interaction, were entered in the analyses described below.

## Results

**Figure 2:**
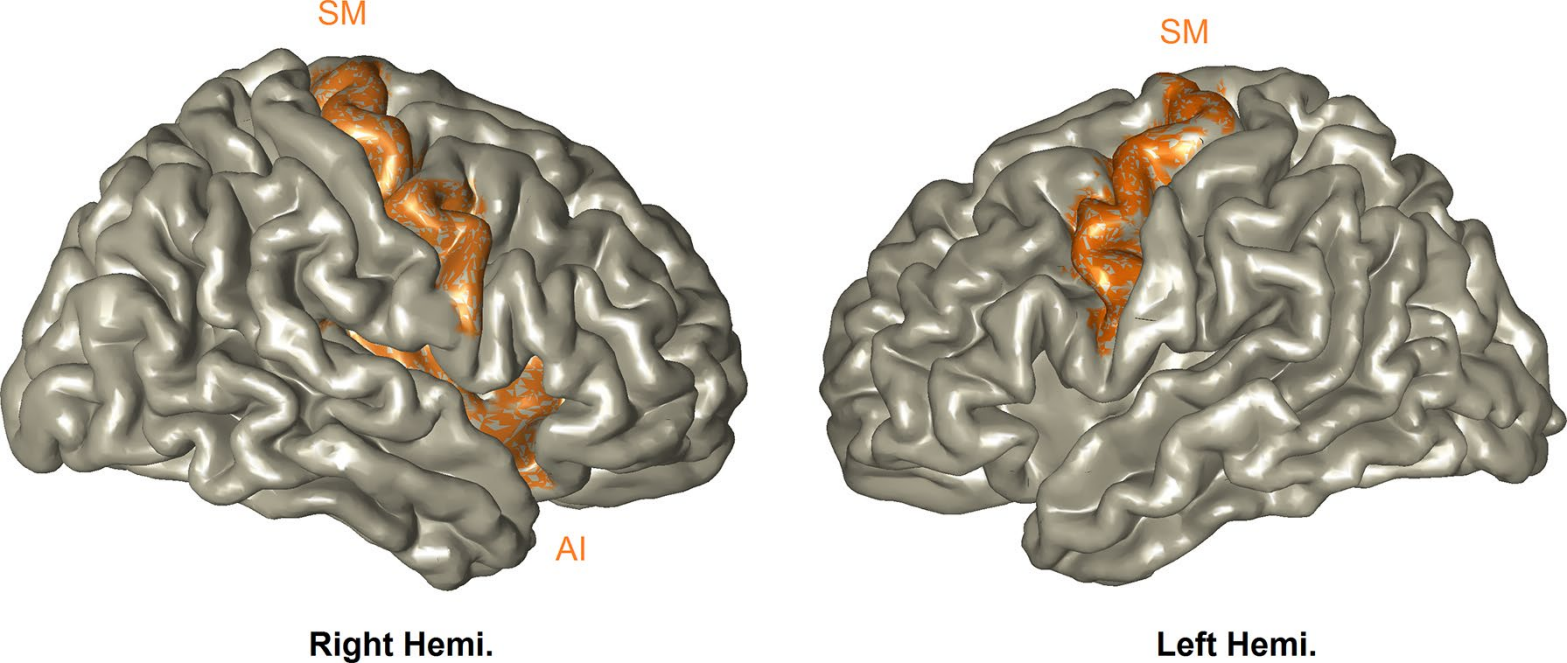
Perception of vicarious distress. All participants activated the bilateral SM and right-AI (*P*_cluster- corrected_ < 0.001), and this activation was expressed as alpha-band suppression.

***Neuroimaging Results.*** We first contrasted stimuli involving DS versus no-DS. The statistical time–frequency contrast (0–2 sec; 6–14 Hz) of all MEG sensor-array yielded alpha suppression peaking between 7–11–Hz and 300–850–ms (*P*_cluster-corrected_ < 0.001). Source localization revealed that this activation pattern peaked in the SM-AI, that is, the bilateral SM and the right AI (*P*_cluster- corrected_ < 0.001); the network is simulated in **Figure 2**. This confirms with our expectation that the paradigm used here will trigger both sensory and affective components of empathy. One mother scored more than 3SD above the SM-IA mean, and was considered an outlier and therefore was removed from the ensuing analyses. To explore whether trauma may directly impact this neural response, we compared between the two groups; however, no statistically significant difference emerged (*t*_(85)_ = 1.37, P = .17).

**Figure 3:**
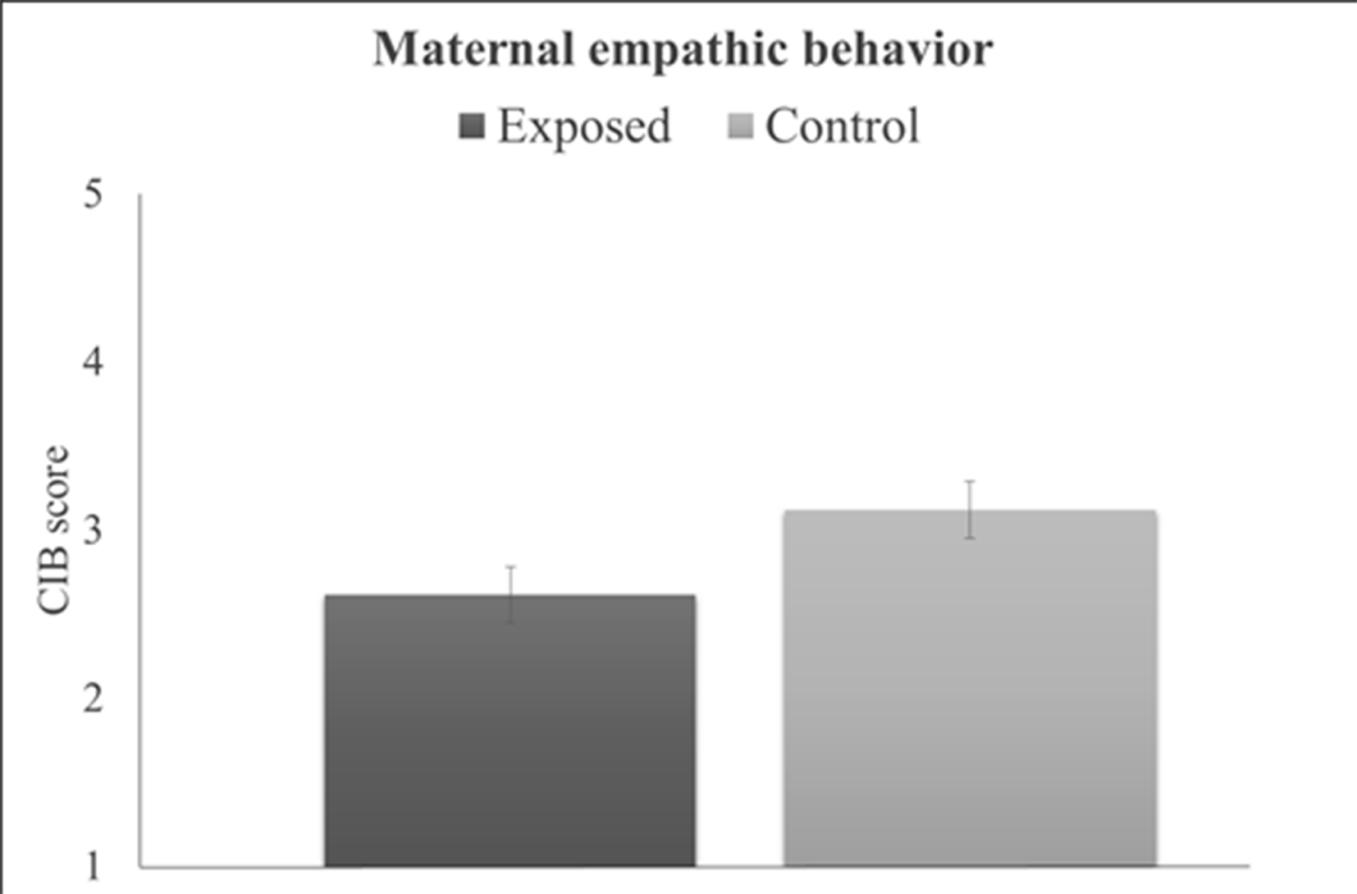
Differences between groups in maternal empathic behavior. Error bars represent ±1 SEM.

***Behavioral Results.*** Maternal behavioral empathy showed a significant group effect (*t*_(77)_ = 2.09, P = .04), with war-exposed mothers displaying less empathy toward their child during the joint task compared to controls (**Figure 3**). There was no significant correlation between maternal empathic behavior and maternal brain activity (r = –.05, P = .66). Results from the PROCESS moderation analyses are displayed in **Table 1** and goodness of fit measures indicated 11% explained variance (R^2^ = .11, *F*_(3,75)_ = 3.03, P = .02). Furthermore, the interaction term added unique explained variance in the model: the change between the model without the interaction effect to the model with the interaction effect was significant (ΔR^2^ = .09, F_(1,75)_ = 8.22, P < .01). No significant main effect of group or maternal behavior emerged for SM-AI. There was, however, a significant group-by-maternal behavior interaction predicting SM-AI brain activity (**Table 1**). For a significant interaction, PROCESS provides the conditional effects of the independent variable at each value of the moderator (i.e., “simple slopes”). As displayed in **Table 1**, tests of simple slopes (54, 55) showed that for control mothers the relationship between maternal empathic behavior and neural empathy (SM-AI) was significant, indicating a negative association between empathic behavior and brain activity (*b* = –.03, *se* = .01, P = .03, β = –.32, *95% CI*: (–.05, –.002)). Taking into consideration that alpha suppression indexes the degree of neural activity, the findings point to a positive association between greater maternal empathic behavior and increased neural activations. In contrast, no significant brain–behavior associations emerged for the trauma-exposed group [*b* = .03, *se* = .01, β = .31, P = .06, *95%CI* (−.001,.05)]. This finding suggests that chronic trauma decouples the links between the neural basis of empathy and empathic parenting behavior in mothers who are raising their children in the context of chronic stress and trauma.

**Table 1:** The moderation effect of exposure and maternal empathic behavior on maternal SM-AI.

Predictor	b(se)	***β***	t	P	95%CI
Exposure	−.02(.02)	−.11	−.93	.36	(−.06,.02)
Maternal behavior	−.003(.009)	−.02	−.28	.78	(−.02,.02)
Exposure maternal behavior	.05(.02)**	.32	2.87	.005	(.02,.09)
Constant	.000(.01)		.04	.97	(−.02,.02)

** P < 0.05.

## Discussion

Previous studies showed that chronic adversity carries dire consequences for the individual’s mental and physical health (27). In the present study, we examined the effects of repeated trauma on the neural and behavioral aspects of empathic abilities. Several important findings emerged from our study, which targeted mothers who are raising their children in the context of chronic trauma exposure. As expected, we found that the neural basis of empathy to the emotional DS of others integrates both sensory and affective processes, as can be inferred by the robust recruitment of both the SM and AI substrates. We have previously shown that the integration of this network is a sign of developmental maturity and is only observed in adulthood, not during childhood or adolescence (12). Yet, contrary to our expectation, we found that chronic trauma did not impair the mothers’ neural empathic response or dampened the integration of the sensorimotor with the interoceptive networks in the maternal brain. However, consistent with our hypotheses, results indicate that exposure to chronic war-related stress decreased the mother’s empathic behavior during interaction with her child and decoupled the empathic brain from the mother’s empathic behavior. Whereas among controls, the mother’s neural empathic response to others’ affective DS was linked to her cognitive, affective, and behavioral empathic response to her child, no such brain-behavior link was found for the trauma-exposed group. As millions of mothers must raise children in conditions of war, terror, and violence, while many others live in dangerous neighborhoods, poverty, and food insecurity, our findings highlight the effects of such contexts on mothers, including the dampening of the mother’s empathic parenting and the diminished coherence between the mother’s social brain and expressed social behavior.

The network of activation found here in response to the emotional DS of others, which includes the SM and AI, was similar to the network of activation we found in a previous study in response to others’ physical pain (12). This implies that empathic neural response to salient bottom-up stimuli describing physical pain is similar to the empathic activations to cognitive and affective top-down stimuli that index emotional pain. An fMRI study that addressed the comparison in the brain response to physical and emotional pain showed similar activations; yet whereas pain empathy activated more sensory and embodied simulation regions including mainly the somatosensory and motor cortices, affective empathy additionally activated higher-order regions, particularly the viceromotor cortex (13). Previous studies provided sound evidence for the role that experimental paradigms of empathy have in the neural activations that they induce. For instance, empathy for pain relies on SM cortices when it is probed by provoking visual stimuli, whereas it relies on higher-order cortices when it is probed by abstract stimuli (5, 6). The present study is in line with this view as it provides empathy which relies on both SM and AI and is probed by both visual and abstract cues.

We used MEG to assess the neural basis of empathy to others’ emotional DS, which uniquely taps the complexity of rhythmic neural activity involved in social and affective experiences in combination with its underlying cortical generators (56) and can therefore provide a different look on the empathic brain as compared to the BOLD (blood-oxygen-level-dependent) signal. Much research has shown that the mu rhythm, the suppression of alpha oscillations above central sensors, provides a good index of the brain’s empathic response (7, 10). Consistent with these studies, the current study, alongside our previous study (12), demonstrate in a combined large sample, perhaps the largest sample in MEG research on the neural basis of empathy, that alpha rhythms are implicated in the two types of empathy; the more automatic empathy to the physical pain of others and the more cognitive and affective empathy to the emotional DS of others. Thus, alpha over central areas participates in the two aspects of empathy, namely, the bottom-up sensory and top-down cognitive/viceromotor processes. Much further research is needed to determine whether the difference between those two components of empathy is expressed by other aspects of neural activity, for instance, neural communication or other neural rhythms in addition to the alpha rhythm.

Viceromotor regions, including the AI, anterior cingulate cortex, and orbitofrontal cortex, are crucial for the perception and understanding of vicarious affect and higher-order empathic representations (2). The mechanisms which are proposed to sustain the viceromotor recruitment suggests that interoception of one’s own body milieu is crucial for empathizing with others (57). We previously showed that viceromotor recruitment is a sign of neural developmental maturity of empathy (12). Likewise, interoception is disrupted in various psychiatric conditions (58); for instance, interoceptive failure is associated with autism spectrum disorder (59) and heightened interoception may be related to anxiety disorders (60), which often result from exposure to trauma (61). These lines of research may be taken as an indication that empathic viceromotor function is a sign of healthy empathic behavior. In the present study we found that exposure to chronic stress does not directly affect the extent of viceromotor activations, but instead, indirectly impairs the correspondence between the empathic expressions of brain and behavior. This is an interesting and innovative finding which deserves further investigation in future research that addresses the links of brain to behavior in health and in cases of various psychopathologies.

The present study replicates prior studies on the neuroscience of empathy but at the same time adds to the emerging literature on the effects of trauma on empathy. One important aspect that the present study raises is the correspondence between the neural and behavioral manifestations of empathy, and the findings that chronic trauma and stress may impair this association. Yet, we recognize one limitation of the present study—it does not provide empirical evidence which could attempt to explain what exactly impairs the correspondence between brain and behavior. One possible explanation pertains to the impairment in emotion regulation which occurs at the neural circuitry and behavioral levels following trauma (28). Given that emotion regulation bridges between targeted neural activity (i.e., sustaining emotions) and its downstream implications (i.e., behavior) (62), it could be that the effects observed here stem from impaired emotion regulation. It would be informative in the future to explore this or other hypotheses to potentially reveal convincing mechanisms responsible for the impaired association observed here. Another shortcoming regards the heterogeneous exposure to trauma across participants, a limitation which is a downside of studying adversity under natural and ecological settings. Although all exposed participants lived in the same frontline neighbourhoods, it is impossible to rule out subtle differences in exposure (e.g., proximity to disaster), which may have resulted in altered neural impact, as previously reported (63).

Although the present study did not directly test for clinical interventions, we propose that the current study can be channeled toward translational venues in future research. The results of the present study demonstrate that stress-exposed mothers do not display the normal association between empathic brain activity and behavior, although their brain activity in of itself does not differ from that of controls, suggesting that exposure to chronic stress impairs empathic abilities mainly in the context of parenting. Based on these findings we suggest that in order to strengthen parenting-related emphatic abilities, a mother–youth brief intervention would be beneficial, and that the effects of such intervention can be measured by the restoration of the association between the mother’s empathic brain activity and her behavior. Previous research assessed the ability of a short-term caregiver–child intervention to prevent the development of PTSD following exposure to a potentially traumatic event (64). However, the effects of such an intervention were studied in a relatively short period following the discrete traumatic event and focused only on the risk of PTSD in the child. We suggest that in situations of chronic exposure to stressful environments, an intervention comprising several sessions which highlight the importance of the maternal acknowledgement of the child’s expressions and needs and DS and sensitive responsivity may improve the mother’s emphatic behavior toward her child. The intervention may involve the mother–child dyad, only the mother, or a combination of these settings, while emphasizing the importance of the mother–child relationship and the well-being of the family in stressful situations. Furthermore, an interesting follow-up study would be to evaluate the brain response and behavior before and after such intervention, assessing whether the association between the mother’s brain activity and empathic behavior increases following the interventions. We recently showed that targeted psychological interventions have the potential to impact empathy at the biological and behavioral levels (65). Thus, a possible take-home recommendation from the present study is that interventions targeting chronic exposure to trauma directly probe the various aspects of empathy: affect, cognition, and behavior.

Finally, our findings are not only relevant to the literature on stress, trauma, and empathy, but tap a more global question: Do brain and behavior always correspond with each other? The brain–behavior correspondence problem stems from the fact that the two operate in different dimensions and are not perfectly matched (66). To the best of our knowledge, this question has rarely been raised before in a systematic empirical program, yet it is important to test whether under certain contexts, including psychopathy or trauma, a dissociation between brain and behavior are more likely to occur. As such, the associations among trauma, brain, and behavior are not only important and uninvestigated topics for future research, but may open new directions to understand the effects of psychiatric conditions on the human social brain.

## Author Contributions

JL, AG and RF designed the study, JL conducted MEG research and analyzed the neural data. KY conducted behavioral assessment and coding and analyzed the data. RF, JL, and KY wrote the manuscript.

## Funding

The work was supported by a NARSAD Young Investigator Grant from the Brain & Behavior Research Foundation to JL, by the award of an Azrieli Fellowship from the Azrieli Foundation to KY, and by grants to RF from the Irving B. Harris Foundation, the Simms/Mann Foundations, and the NARSAD independent investigator award, and by the I-CORE Program of the Planning and Budgeting Committee and the Israel Science Foundation (grant no. 51/11).

## Conflict of Interest Statement

The authors declare that the research was conducted in the absence of any commercial or financial relationships that could be construed as a potential conflict of interest.

## Footnotes

1 Children’s brain was also imaged and results will be presented elsewhere.
